# Antibiotic development challenges: the various mechanisms of action of antimicrobial peptides and of bacterial resistance

**DOI:** 10.3389/fmicb.2013.00353

**Published:** 2013-12-09

**Authors:** Fernanda Guilhelmelli, Nathália Vilela, Patrícia Albuquerque, Lorena da S. Derengowski, Ildinete Silva-Pereira, Cynthia M. Kyaw

**Affiliations:** ^1^Laboratório de Biologia Molecular, Departamento de Biologia Celular, Instituto de Ciências Biológicas, Universidade de BrasíliaBrasília, Brazil; ^2^Laboratório de Microbiologia, Departamento de Biologia Celular, Instituto de Ciências Biológicas, Universidade de BrasíliaBrasília, Brazil

**Keywords:** antimicrobial peptides, mechanism of action, mechanism of resistance, bacterial, membrane permeability and intracellular targets

## Abstract

Antimicrobial peptides (AMPs) are natural antibiotics produced by various organisms such as mammals, arthropods, plants, and bacteria. In addition to antimicrobial activity, AMPs can induce chemokine production, accelerate angiogenesis, and wound healing and modulate apoptosis in multicellular organisms. Originally, their antimicrobial mechanism of action was thought to consist solely of an increase in pathogen cell membrane permeability, but it has already been shown that several AMPs do not modulate membrane permeability in the minimal lethal concentration. Instead, they exert their effects by inhibiting processes such as protein and cell wall synthesis, as well as enzyme activity, among others. Although resistance to these molecules is uncommon several pathogens developed different strategies to overcome AMPs killing such as surface modification, expression of efflux pumps, and secretion of proteases among others. This review describes the various mechanisms of action of AMPs and how pathogens evolve resistance to them.

## ANTIMICROBIAL PEPTIDES

Antimicrobial peptides (AMPs), an important part of the innate immune system, are small molecules that may present antibacterial, antifungal, antiparasitic, and antiviral activity (Hancock and Diamond, 2000; Jenssen et al., 2006). Usually these molecules are composed of 10–50 amino-acid residues, and arranged in different groups depending on the amino-acid composition, size, and conformation (Lai and Gallo, 2009; Nakatsuji and Gallo, 2012).

The largest group corresponds to cationic peptides, which is divided in three classes (Vizioli and Salzet, 2002; Brogden, 2005). The first class is composed by linear cationic α-helical peptides, such as Magainin and Cecropins that are linear before their interaction with the cell membrane, and then adopt an amphipathic α-helical secondary structure (Bechinger et al., 1993). The second class comprises cationic peptides enriched for specific amino acids like proline, arginine, and other residues. These peptides are linear, although some may exhibit extended coils (Brogden, 2005). The third class includes cationic peptides that contain cysteine residues and form disulphide bonds and stable β-sheets. Defensins, an example of this class, have six cysteine residues and are divided according to the alignment of their disulphide bridges (α-, β-, and θ-defensin) (Mehra et al., 2012).

Other groups of AMPs are described as non-cationic peptides, anionic peptides, aromatic peptides, and peptides derived from oxygen-binding proteins (Vizioli and Salzet, 2002). Just as the cationic peptides, anionic peptides (AAMP) are also an important part of the innate immune system and have been identified in vertebrates, invertebrates, and plants (Harris et al., 2009). Although the antibacterial activity of these peptides is considered weak, they could improve the activity of cationic peptides (Vizioli and Salzet, 2002).

Different studies revealed that besides peptide charge (cationic or anionic), other characteristics such as size, primary sequence, conformation, structure, hydrophobicity, and amphipathicity could be essential for antimicrobial activity and mechanism of action (Friedrich et al., 2000; Gennaro and Zanetti, 2000).

## MAIN MECHANISMS OF ACTION

The classic action mechanism of AMPs involves their ability to cause cell membrane damage. AMPs can interact with microorganisms by electrostatic forces between their positive amino acid residues and negative charges exposed on cell surfaces. It has been suggested that the composition of cell surface drives the specificity of AMPs. In this sense, the sensitivity of prokaryotic and eukaryotic cells is directly related to the different physicochemical properties of the lipids found on both cell membranes (Dathe and Wieprecht, 1999; Matsuzaki, 1999). In mammalian membranes, the lipids most commonly found on the extracellular side of the bilayer are neutral phospholipids such as phosphatidylcholine and sphingomyelin. On the other hand, the bacterial cell membrane is essentially composed by negatively charged lipids, such as phosphatidylglycerol (PG), cardiolipin, and the zwitter ionic phosphatidylethanolamine (PE) (Lohner, 2009). The overall negative charge found on the membrane of bacteria has an important role in the preferential binding of some peptides to those microorganisms. Moreover, Gram-negative bacteria contain lipopolysaccharides (LPS) on their outer membrane, and the cell wall of Gram-positive bacteria is enriched in acidic polysaccharides (teichoic and teichuronic acids). Those molecules that confer a negative charge to the bacterial surface were selected as targets for cationic AMPs (Brogden, 2005).

The interaction and action of AMPs with their target cells depends largely on the cell surface as well as on the amino acid composition of these peptides. This idea is supported by the high conservation of positively charged amino acid residues among peptides sequences from various organisms (Yeaman and Yount, 2003). In addition, the secondary structure adopted by the peptide is essential for the binding to negatively charged compounds in the target membrane, such as anionic phospholipids (Matsuzaki, 2009). Depending on the peptide/lipids ratios and affinity, these peptide molecules can be oriented perpendicularly, allowing their insertion into the lipid bilayer and the formation of transmembrane pores (Brogden, 2005; Melo and Castanho, 2012).

The mechanisms by which AMPs can traverse microbial membranes are not common to all peptides and seem to depend on the molecular properties of both, peptide addressed and lipid membrane composition. Several membrane defects can be induced by AMPs, among them we can highlight formation of pores, phase separation, and promotion of non-lamellar lipid structure or disruption of the membrane bilayer (Lohner and Prenner, 1999). Some models that may explain membrane disruption by AMPs have been proposed, such as barrel-stave, toroidal, and carpet models.

The first mechanism proposed, the “barrel-stave” model, suggests that peptides form transmembrane pores by their direct insertion into the lipid core of the target membrane (Ehrenstein and Lecar, 1977). In this model, AMP binds to the membrane surface as a monomer, which is followed by their oligomerization and pore formation. The recruitment of additional monomers can increase the pore size, allowing cytoplasmic content leaking with subsequent cell death. In this mechanism, peptide secondary structures, such as hydrophobic α-helix and/or β-sheet, are essential to pore formation (Breukink and de Kruijff, 1999). These peptide regions interact with the membrane lipids, while the hydrophilic peptide regions form the lumen of the channel (Brogden, 2005).

In contrast to the “barrel-stave” model, in the “toroidal” model, peptide molecules are inserted into the membrane forming a bundle, inducing the lipid monolayers to continuously bend through the pore (Yang et al., 2001). As a consequence, membrane lipids become interspersed with peptides forming the pore (Yeaman and Yount, 2003). Examples of AMPs toroidal pore forming are megainins, protegrins, and melittin (Brogden, 2005).

According to the “carpet” model, AMPs cover the membrane surface affecting its architecture, in a detergent-like manner (Rotem and Mor, 2009). Their interaction is first driven by electrical attraction and, when the amount of AMPs on the membrane surface reaches a threshold concentration, the membrane disintegrates, leading to cell lysis (Oren and Shai, 1998). However, it was described that some peptides might also form transmembrane pores at concentrations below the threshold, suggesting that the mechanism by which the peptide disrupts/permeates the membrane depends on its concentration (Lohner, 2009).

Although most AMPs interact directly with cell membrane lipids, it was reported that some peptides might require a bacterial receptor. This hypothesis was supported by the fact that the antimicrobial activities of the all-L and all-D enantiomers of some peptides, such as apidaecin and drosocin, are not the same (Casteels and Tempst, 1994; Bulet et al., 1996).

More recently it has been proposed that AMP driven microbial death can be caused by others mechanisms in addition to membrane disruption, followed by cell lysis. Many evidences indicate that some AMPs can interact with intracellular targets inducing cell damages, such as the inhibition of cell wall, DNA, RNA, and protein synthesis (Brogden, 2005; Straus and Hancock, 2006). Furthermore, one peptide can act upon multiple cell targets, involving a mixed multi-hit mechanism (Jenssen et al., 2006; Straus and Hancock, 2006). Finally, it is important to note that AMPs action may vary according to test conditions and can be influenced by external factors such as media pH, osmolarity, and temperature (Yeaman and Yount, 2003).

## INTRACELLULAR TARGETS

### INHIBITION OF CELL WALL SYNTHESIS

The cell wall is an essential bacterial structure responsible for the cell shape. Additionally, the cell wall prevents cell lysis due the high cytoplasmic osmotic pressure and allows the anchoring of membrane components and extracellular proteins, such as adhesins. In Gram-positive organisms, the main component of the cell wall is the peptidoglygan, present in multiple layers. In Gram-negative bacteria, an outer membrane, composed mainly by LPS, overlaps a thin layer of peptidoglycan. Since peptidoglycan is not found in eukaryotic cells, compounds that inhibit its synthesis are interesting targerts for therapeutics. In particular, lipid II, which is an important precursor of peptidoglycan synthesis, was shown to be an attractive target of antibacterial compounds, such as several lantibiotics (de Kruijff et al., 2008; Yount and Yeaman, 2013).

Class I bacteriocins, also known as lantibiotics, are a family of AMPs ribosomally synthesized and post-translationally processed to produce numerous molecules with unusual modified residues (Yount and Yeaman, 2013). Besides killing bacteria by targeting lipid II, and consequently inhibiting cell wall biosynthesis, it has been described that the lantibiotics can also form transmembrane pores, that allows the efflux of molecules and/or ions – ATP, K^+^, and PO_4_^3^^-^ (Islam et al., 2012). Several studies have demonstrated that nisin, an antimicrobial peptide widely used in food industry, first binds to lipid II and then acts by forming pores in the membrane, with consequent loss of amino acids, K^+^, and ATP by the cell (Yount and Yeaman, 2013). A recent study also demonstrated that nisin binds to lipids III and IV, interfering with teichoic and lipoteichoic acids biosynthesis (Muller et al., 2012). In addition, nisin appears to stimulate the activity of the autolysin *N*-acetylmuramoyl-L-alanine amidase, as shown in *Staphylococcus simulans*. Similar stimulatory effect on this autolysin activity was observed using Pep5, an antimicrobial peptide produced by *S. epidermidis* (Bierbaum and Sahl, 1987). The induction of autolysins might result in cell wall damage, leading to lysis and subsequent cell death.

Mersacidin, another well-characterized lantibiotic, is effective against methicillin-resistant *S. aureus* (MRSA) in a murine infection model (Chatterjee et al., 1992; Kruszewska et al., 2004). The binding site of mersadicin to lipid II differs from that described for nisin: while nisin binds to the phosphate, mersacidin probably binds to sugar residues. Despite of the different binding sites, both AMPs inhibit the formation of the peptidoglycan (Brotz et al., 1998;Islam et al., 2012).

Another group of lantibiotics is the two-peptide family, whose members are composed by two independently transcribed peptides that act synergistically in order to have an optimal antimicrobial activity (Lawton et al., 2007; Yount and Yeaman, 2013). It has been proposed that one of the peptides binds to lipid II while the other forms transmembrane pores (Wiedemann et al., 2006). Lacticin 3147, staphylococcin C55, plantaricin W, and haloduracin are examples of the two-peptide lantibiotic family (Lawton et al., 2007).

In addition to lantibiotics, other AMPs target the cell-wall synthesis. Lcn972, is a representative of class II bacteriocins that probably exerts an antimicrobial activity via binding lipid II, diverging from the most of other peptides from its class, that act preferentially via membrane disruption. Similarly, nonribosomally synthesized peptides, such as vancomycin, daptomycin, telavancin, and dalbavancin, as well as some defensins produced by eukaryotic organisms, target lipid II biosynthesis pathway, acting via a cell-wall inhibitory mechanism (Yount and Yeaman, 2013).

Because of the main role of bacterial cell wall in cell integrity, as well as its absence in mammalian cells, this structure becomes an excellent target in the search of new antimicrobial drugs. Moreover, the combinatory mechanisms of cell wall synthesis inhibition and cell membrane disruption observed for some AMPs could minimize the emergence of resistant microorganisms.

### INHIBITION OF NUCLEIC ACID AND PROTEIN SYNTHESIS

Some AMPs can spontaneously traverse bacterial outer and inner membranes, targeting intracellular molecules such as nucleic acids and proteins. Buforin II, a 21 amino acid, cationic and linear molecule, is an example of an AMP able to cross the cell membrane without permeabilizing it, which later accumulates in the cytoplasm (Cho et al., 2009).

It is well known that this peptide has a proline hinge (Pro^11^) that plays a critical role in promoting the peptide penetration into the cell (Cho et al., 2009; Xie et al., 2011). Once in the cytoplasm, buforin II binds to DNA and RNA, as showed for *Escherichia coli* (Park et al., 1998). The strong affinity of this peptide for nucleic acids *in vitro* (Park et al., 1998) might be explained by the sequence identity between buforin II and the N-terminal region of histone H2A (Cho et al., 2009). Curiously, buforin II has an antiendotoxin activity (Giacometti et al., 2002). The significant reduction of endotoxin plasmatic levels mediated by buforin II is remarkable since the endotoxin released in Gram-negative infections is responsible for strong inflammatory reactions, with the production of tissue-damaging cytokines, resulting in multiple organ failure and, sometimes, host lethality (septic shock syndrome). The capability to neutralize the toxic effects of endotoxin, preventing the septic shock, has been described for many other AMPs, such as temporins (Mangoni and Shai, 2009) and cathelicidins (Mookherjee et al., 2007).

Many other AMPs act by inhibiting nucleic acid synthesis. Indolicidin, one of the smallest natural cationic peptide, appears to act promoting significant membrane depolarization and inhibiting DNA synthesis (Subbalakshmi and Sitaram, 1998; Nan et al., 2009). As a consequence of the inhibitory effect on DNA synthesis, indolicidin induces filamentation of *E. coli* cells (Subbalakshmi and Sitaram, 1998).

Puroindoline, similarly to indolicidin, is a family of peptides rich in tryptophan residues, that also act by inhibiting DNA synthesis. Haney et al. (2013), using radioactive precursors for DNA, RNA, and protein biosynthesis, demonstrated that the PureB, a peptide of the puroindoline family, inhibits the transcription process and, therefore, the translation process. However, it is still unknown how indolicidin and puroindoline bind to the nucleic acid. Probably, the positive charges of these peptides interact with the phosphate groups of the nucleic acids (Park et al., 1998; Uyterhoeven et al., 2008).

Cathelicidins comprise a family of mammalian proteins with a wide spectrum of antimicrobial activity. One representative is PR-39, which is involved in several cellular processes, including wound repair, chemoattraction, angiogenesis, and inflammation (Zanetti, 2004; Kaneider et al., 2007). This AMP rapidly crosses the cell, without causing membrane damage, and blocks DNA and protein synthesis in bacteria (Boman et al., 1993; Chan et al., 2001; Bals and Wilson, 2003). In addition, some studies propose that PR-39 acts as a noncompetitive and reversible inhibitor of the 20S proteasome, blocking the degradation of NF-κB inhibitor, IκBα. As a result, NF-κB–dependent gene expression is suppressed thereby attenuating inflammation (Gao et al., 2000; Anbanandam et al., 2008). The multiple effects of PR-39 in the target cell might reflect its ability to selectively bind to cytosolic proteins containing SH3 domains (Kaneider et al., 2007).

Another family of cationic peptides with potent antimicrobial activity is the bactenecins, present in bovine neutrophil granules. Two members of this family, Bac5 and Bac7, act by increasing membrane permeability and inhibiting protein and RNA synthesis in *E. coli* and *Klebsiella pneumoniae* (Skerlavaj et al., 1990). Human neutrophils also produce large amounts of AMPs, such as defensins. Besides the ability to permeabilize cell membranes, the human defensin HPN-1 inhibits nucleic acid and protein synthesis in *E. coli* (Lehrer et al., 1989). More recently it was proposed that this AMP also targets the cell wall synthesis by binding to lipid II precursor (de Leeuw et al., 2010). These data support the idea that a single AMP can have multiple mechanisms of action that simultaneously contribute for microorganism death.

Microcins, a group of AMPs produced by some enterobacteria, can also inhibit intracellular targets. Microcin C (McC) is a non-hydrolyzable aminoacyl adenylate that is imported into bacterial cells by an outer-membrane porin and an inner-membrane ABC (ATP-binding-cassette) transporter. Once in the cytoplasm, McC inhibits an essential aminoacyl-tRNA synthetase (Nocek et al., 2012; Rebuffat, 2012). Microcidin B17 is a modified bacterial peptide that targets DNA gyrase, with subsequent inhibition of DNA replication (Collin et al., 2013). A single mutation in DNA gyrase seems to be enough for bacterial resistance (del Castillo et al., 2001). Microcidin B17 penetration into bacterial cell is facilitated by the inner-membrane protein SbmA (Mathavan and Beis, 2012), which also helps microcidin J25 to cross the cell envelope, where it binds to the RNA polymerase, inhibiting bacterial transcription (Mathavan and Beis, 2012; Rebuffat, 2012). Despite using different mechanisms of action, most microcins employ the “Trojan-horse” strategy, interacting with a bacterial component that facilitates their active transport into the cell. Once inside de cell, the transport component is removed, and the microcin peptide is released to bind to its target (Rebuffat, 2012; Severinov and Nair, 2012).

### INDUCTION OF CELL DEATH

Cell death occurs mostly in a programmed form, known as apoptosis, or in an unordered and accidental manner, known as necrosis (Kanduc et al., 2002). Although some mechanisms that cause the cell death are not clear yet, the importance of the caspase cascade in the induction of apoptosis is well known (Fink and Cookson, 2005; Chowdhury et al., 2006). These proteases, in particular caspase-3, catalyze reactions that induce a rapid disarrangement in signaling, homeostatic and repair enzymes, killing the cell (Nicholson and Thornberry, 1997).

Other signals have been described to provoke apoptosis and other mechanisms of death. Bortner and Cidlowski report that, when the concentration of intracellular potassium (K^+^) is normal, the cell death process is repressed by the suppression of the caspase cascade and inhibition of the apoptotic nucleases activity. Thus, the efflux of K^+^ could prompt cell apoptosis (Bortner and Cidlowski, 1999). In the same way, calcium ions (Ca^2^^+^) and reactive oxygen species (ROS) work as pro-apoptotic second messengers (Chowdhury et al., 2006). These signals influence the mitochondrial homeostasis that activate the caspase cascade by releasing the cytochrome *c* (Li et al., 1997).

Currently, the number of studies in this area has increased due to the high frequency of various types of cancer. In this sense, the comprehension of these signals could improve the development of anti-tumoral drugs that could prevent the growth of tumors. Several reports have described antitumor activity for different AMPs. Magainin, a cationic peptide, induces apoptosis by increasing the levels of ROS and the caspase-3 activity in HL-60 cells, a cancer cell line from acute promyelocytic leukemia (Cruz-Chamorro et al., 2006). With the same cell line, other peptide, tachyplesin, that has a disulphide brigde and is stabilized in a β-hairpin structure, induces apoptosis by modifying the potassium intracellular concentration independent of caspase activation (Zhang et al., 2006).

With an erythro-leukemia cell line (K562), four β-hairpin AMPs (gomesin, protegrin, tachyplesin, and polyphemusin II) were tested and, despite of having the same structure, these peptides showed different intracellular mechanisms of cell death induction (Paredes-Gamero et al., 2012). Gomesin, for example, induce K562 cells apotosis by phosphatidylserine externalization and caspase-3 activation, with no cell membrane permeabilization (Paredes-Gamero et al., 2012), while in B16 melanoma, SH-SY5Y neuroblastoma and PC12 pheochromocytoma cells it causes apoptosis inducing Ca^+^-dependent membrane permeabilization (Soletti et al., 2010). On the other hand, protegrin provokes cell death inducing a necrosis process by the increase of Ca^+^levels (Paredes-Gamero et al., 2012). Paredes-Gamero et al. also demonstrated that the mechanism of action of these four β-hairpin AMPs depend on their concentration correlated to their EC_50_values. At low concentrations these peptides induced different intracellular mechanisms of cell death, as mentioned above. However, at high concentrations, these peptides interact with the membrane and form pores, killing the cell. The mechanism of its interaction to mammalian cell surface is not clear and does not fit to the models described above, since eukaryotic cells are quite different from bacterial cells. Finally, this corroborate that the structure of the peptide is not the only factor that determines the mechanism of action in a cell. Furthermore, the concentration used in an experiment could change the peptide interaction with the cell target and its death.

Similar to these antitumor peptides, some antifungal peptides could also induce fungal cell death by apoptosis, necrosis, or other mechanisms (van der Weerden et al., 2013). PvD(1), a plant defensin from *Phaseolus vulgaris* seed, is active against *Candida albicans* and *Fusarium oxysporum*, a human and a plant pathogen, respectively (Mello et al., 2011). This peptide kills the fungal cell by membrane permeabilization and the induction of oxidative stress damage, with the production of ROS and nitric oxide (NO; Mello et al., 2011). The link between endogenous ROS levels and apoptosis is also described to another plant defesin HsAFP1 (Aerts et al., 2011). This peptide could be found in coral bell seeds (*Heuchera sanguinea*) and inhibits several pathogenic fungi, such as *C. albicans* (Osborn et al., 1995; Thevissen et al., 2007). Although the membrane target has not been identified yet, but inside the cell this defensin induces several pro-apoptotics signals, such ROS accumulation, phosphatidylserine externalization, and DNA fragmentation, provoking cell death (Aerts et al., 2011).

To better understand the resistance against AMPs, in the next section, we will focus on this important issue.

## MAIN MECHANISMS OF RESISTANCE TO AMPS IN BACTERIA

As discussed in the previous section we are now experiencing a rapid increase in the number of alternative mechanisms for AMPs microbial killing, showing that these molecules are more versatile than we have imagined. On the other hand, in spite of the crescent number of described AMPs active against multiple species of pathogenic organisms, several studies are also showing different strategies of natural or induced resistance to AMPs among different bacterial species, pointing us to the co-evolution of AMPs and AMP-resistance mechanisms (Peschel and Sahl, 2006). Although fungal species also present mechanisms of AMP resistance in the following sections we will focus on mechanisms of resistance in bacteria. In general, AMP resistant bacteria have developed multiple and frequently overlapping strategies of resistance, normally controlled by coordinated stress responses regulated by operons. The most common resistance mechanisms involve changes in bacterial cell surface and blockage of the AMP access to their targets by affecting its binding and/or its penetration into the cells and will be discussed in the following sections.

### SURFACE REMODELING

Gram-positive and Gram-negative bacteria modify their cell wall components to reduce the net negative charge of their surfaces. One strategy in Gram-negative bacteria is masking the negative charge of the LPS lipid A by adding amine-containing compounds such as ethanolamine and 4-amino-4-deoxy-T-arabinose (Ara4N). It has been described that differences in polymyxin susceptibility between *Vibrio cholerae* O1 El Tor and classical biotypes could be explained by the ability of *V. cholerae* O1 El Tor to modify the lipid A anchor with glycine and diglycine residues (Hankins et al., 2012). These authors have also shown that the enzymes required for glycine addition are similar to the Gram-positive system for D-alanylation of teichoic acids. In addition to the binding of Ara4N to phosphates of lipid A, palmitoylation of lipid A also promotes resistance to AMPs. *Salmonella* sp., *E. coli, Yersinia enterocolitica*, and other enteric bacteria increase lipid A acylation in response to activation of the Pho-*P*-PhoQ regulon. On the other hand, mutants in the pathway responsible for this modification revealed increased permeability to AMPs (Guo et al., 1998).

Interestingly, the surface modification leading to cationic AMP resistance can occur in response to the presence of the cationic AMP itself. *S. enterica* serovar *typhimurium* (*S. typhimurium*) uses the two-component regulatory system PhoP-PhoQ and PmrA-PmrB to sense the presence of environmental factors in the host tissues such as CAMPs, which results in the activation of the enzymes responsible for LPS modification and CAMP resistance. In this case, when activated, the operon regulates the synthesis and attachment of positively charged Ara4N groups to lipid A. The surface of *S. typhimurium* cells growing *in vitro* in LB broth or on plates mainly present unmodified LPS while the highly modified version of LPS is predominant when the cells are grown *in vivo* (Strandberg et al., 2012). PhoP-PhoQ and PmrA-PmrB systems are also activated in *Pseudomonas aeruginosa* in response to AMPs or low concentrations of divalent cations resulting in the resistance to polymyxin B and other CAMPs (McPhee et al., 2003; Moskowitz et al., 2004; McPhee et al., 2006). Another pathogen, *Y. pestis,* also depends on a two-component regulatory system to respond to AMPs, since mutants of the PhoPQ pathway had decreased intracellular survival in PMN (O’Loughlin et al., 2010). Similarly, Pietiainen et al. found that, in *S. aureus*, AMPs induce the expression of two operons VraSR cell-wall regulon and the vraDE operon. Among the genes activated in this response there was an ABC transporter, a possible peptidase and a small unknown protein. The vraDE seemed to be involved mainly in bacitracin resistance, while VraSR was shown to act in the resistance to several cell wall-active antibiotics and other antimicrobial agents including AMPs (Pietiainen et al., 2009).

*Helicobacter pylori* is a Gram-negative bacterium that colonizes the stomach mucosa and can cause peptic ulcer and gastric cancer. The strategy of these bacteria to avoid AMPs consists in the dephosphorylation of the lipid A fraction of LPS followed by the addition of a phosphoethanolamine group (Tran et al., 2006). Cullen et al. showed that *H. pylori* mutants that are unable to remove the phosphate groups are highly sensitive to polymyxin and also more readily recognized by the innate immune Toll-like receptor 4 (Cullen et al., 2011).

The main strategy used by Gram-positive bacteria to reduce the negative charges in their surface is the D-alanylation of teichoic acids, mediated by the *dlt* operon and/or the incorporation of T-lysine into PG by the *mprF* gene product (Kristian et al., 2005; Andra et al., 2011; Saar-Dover et al., 2012). The *dlt* operon mediates the esterification of teichoic acids with D-alanyl esters while the membrane protein MprF modifies PG by the enzymatic transfer of L-lysine that changes the net charge of this lipid to become positive (Neuhaus and Baddiley, 2003; Andra et al., 2011). Mutations in genes of both pathways were shown to drastically increase the susceptibility of several Gram-positive strains to AMPs (Peschel et al., 1999; Kovacs et al., 2006; Thedieck et al., 2006; Abi Khattar et al., 2009; Andra et al., 2011; McBride and Sonenshein, 2011). Interestingly, Saar-Dover et al. suggested that the main mechanism involved in the AMP resistance mediated by the D-alanylation is an increase in the cell wall density impairing the penetration of AMPs instead of a major effect on the AMP binding to bacterial surface (Saar-Dover et al., 2012). However, further studies with other species are necessary to confirm if this mechanism can be generalized.

The work of Shireen et al. (2013) reinforce the idea that alterations in surface net charge are not always the main mechanism of surface modification leading to AMP resistance. They analyzed the effects of sub-lethal concentrations of two AMP, magainin 2 and gramicidin D, on the rise of resistant strains of *S. aureus*, and found that the two peptides induced different resistance strategies that result in an increase of membrane rigidity (Shireen et al., 2013).

Another surface modification that can be related to AMP resistance is capsule production. Capsular polysaccharide may acts as a shield, avoiding the interactions between the microbial surface and CAMPs. For example, Campos et al. (2004) have shown that *K. pneumoniae* capsule makes their cells more resistant to defensins, lactoferrin and polymyxin B, while acapsular mutants bound more AMP than wild-type strains. Interestingly, they also found that CPS expression is induced in the presence of AMPs, as previously observed with antibiotic treatment. Additionally to *K. pneumoniae*, CPS from *Streptococcus pneumoniae* serotype 3 and *P. aeruginosa* also increased resistance of a *K. pneumoniae* acapsular strain to polymyxin and for α-defensin (Llobet et al., 2008). The authors suggest that these bacteria can induce the release of capsular polysaccharide in response to AMPs in order to inhibit their interaction with their targets (Llobet et al., 2008). This phenomenon is also observed with *Neisseria meningitidis* where CPS contributes to LL-37 resistance (Jones et al., 2009). In *Campylobacter jejuni*, however, capsule expression was not essential to mediate AMPs resistance, but only to serum components. In this species, resistance to α-defensins, cathelicidins (LL-37) and polymyxin B seems to be related to lipooligosaccharides produced by this organism, as demonstrated by the increased susceptibility of the truncate lipooligosaccharide mutant, when compared to the wild-type strain (Keo et al., 2011). Shielding of AMP targets is also performed by alginate, a polymer secreted by *P. aeruginosa* during biofilm formation that induces changes in AMP conformation, avoiding their interaction with microbial membrane (Chan et al., 2004).

### MODULATION OF AMP GENES EXPRESSION

The highly contagious and invasive Gram negative rod *Shigella* spp. cause bacillary dysentery that can be often fatal to infants and children. *S. flexneri* was shown to subvert the immune system by down-regulating the expression of innate immune response genes, among them the genes of the cathelicidin LL-37 and the human β-denfensin-1 (Islam et al., 2001; Sperandio et al., 2008). This down-regulation probably facilitates bacterial adhesion and invasion as lower levels of AMPs correlates with deeper invasion of *S. flexneri* toward intestinal crypts (Sperandio et al., 2008).

Several species of the genus *Burkholderia* are inheritably resistant to AMPs. This genus includes several human pathogens among them the *Burkholderia cepacia* complex, which are particularly important for causing chronic opportunistic infections especially in patients with cystic fibrosis or chronic granulomatous disease (Mahenthiralingam et al., 2005; Loutet and Valvano, 2011). One of the explanations for the high resistance of *Burkholderia* sp. to AMPs is the constitutive production of Ara4N as part of their LPS molecule (Cox and Wilkinson, 1991; Isshiki et al., 1998; Loutet and Valvano, 2011). The other strategy used by this group is mediated by the alternative sigma factor RpoE, which controls several genes responsive to stress conditions, however the genes involved in this process are not know yet (Flannagan and Valvano, 2008; Loutet et al., 2011). Interestingly, RpoE was shown to be required for *B. cenocepacia* polymixin B resistance at 37°C, but not at 30°C (Loutet and Valvano, 2011).

Another example of modulation of host AMP production by the pathogen was observed in experiments using *K. pneumoniae* capsular mutant strains. Using acapsular mutants Moranta et al. found that the polysaccharide capsule is also involved in the inhibition of human β-defensins expression. They observed that an acapsular mutant induced the expression of β-defensins by engaging TLR 2 and 4 and activating NF-κb MAPK pathways necessary for β-defensins expression, while the wild-type strain or LPS O antigen mutant did not (Moranta et al., 2010).

### BIOFILMS

Biofilms are microbial communities consisting of cells attached to biotic or abiotic surfaces, embedded in an exopolymeric matrix. These structures are well known for their remarkable resistance to diverse chemical, physical, and biological antimicrobial agents and are one of the main causes of persistent infections. Biofilms are also more resistant to AMPs and this feature is linked, although not restrict, to changes in gene expression of biofilm cells, the nature of exopolymeric matrix, and biofilm architecture.

Chan et al. have shown that alginate, a polysaccharide of *P. aeruginosa* biolfim matrix has an important role in *P. aeruginosa* resistance to AMPs (Chan et al., 2004). First, alginate mimics the microbial membrane by inducing conformational changes in AMPs similar to those that occur during the peptide insertion in the membrane bilayer. In addition, alginate also prevents AMPs diffusion because of its ability to bind and induce peptide aggregation, impairing their action (Chan et al., 2004, 2005).

DNA is also a major component of the biofilm matrix. Mulcahy et al. found that exogenous DNA chelates cations, inducing AMP resistance to aminoglycosides and cationic AMPs in *P. aeruginosa* (Mulcahy et al., 2008). The authors observed that extracellular DNA has antimicrobial activity, but that sub-inhibitory concentrations of exogenous DNA induced microbial resistance. The resistance was partially explained by the ability of DNA to chelate several divalent cations, activating the expression of antimicrobial resistance genes such those involved in LPS modification and also the release of genomic DNA, what can result in a DNA gradient inside biofilms (Mulcahy et al., 2008). Similar results were found in a study with *S. typhimurium*, showing that extracellular DNA also induced the expression of *pmr* genes, resulting an increased resistance to AMPs (Johnson et al., 2013).

Moreover, Folkesson et al. established that the differential organization observed in *E. coli* biofilms due to the presence of the transfer plasmids is crucial for AMP resistance. Bacterial strains lacking the transfer plasmid formed less organized biofilms, which were more susceptible to AMPs. The biofilm resistance was also dependent on a two-component regulatory system homolog to the *pmrA-pmrB*. Interestingly, the resistance was not a biofilm general property, being observed only in a subpopulation of cells within the biofilm, as previously observed for *P. aeruginosa* biofilm resistance to colistin (Haagensen et al., 2007; Folkesson et al., 2008).

### PROTEOLYTIC DEGRADATION

Gram-positive and Gram-negative bacteria produce peptidases and proteases that degrade CAMPs. Linear peptides are the main target for those enzymes, since proteolytic sites are more exposed to cleavage. The human antimicrobial peptide, LL-37, is digested by proteases of many human pathogens, like *P. aeruginosa*, *Enterococcus faecalis*, *Proteus mirabilis*, *S. aureus*, and *S. pyogenes* (Schmidtchen et al., 2002; Sieprawska-Lupa et al., 2004; Johansson et al., 2008). The elastase of *P. aeruginosa* rapidly degrades LL-37, generating early intermediate fragments presenting residual bactericidal activity when incubated with *E. faecalis*. However, incubation with late digestion fragments shows no bactericidal effect, demonstrating the complete degradation and loss of function of LL-37. Notably, elastase preferentially cleaves LL-37 regions involved in its antimicrobial activity (Schmidtchen et al., 2002).

The metalloprotease ZapA produced by *P. mirabilis* is an important virulence factor involved in the degradation of AMPs (Belas et al., 2004). ZapA cleaves the β-defensin hBD1 and LL-37, partially cleaves PG-1, and is incapable to digest hBD2, in spite of the high similarity between hBD2 and hBD1. ZapA also cleaves LL-37 preferentially on sites inside the antimicrobial domains of this cathelicidin, as already described (Schmidtchen et al., 2002). ZapA digestion is not specific to AMPs, since it also degrades other molecules, like cell matrix components (actin, collagen, fibronectin, laminin), antibodies (IgA and IgG), and complement C1q and C3 (Belas et al., 2004). On the other hand, mutants of ZapA are no more susceptible to those AMPs than the wild-type strain, indicating that *P. mirabilis* may use multiple mechanisms to avoid the AMPs action. Similarly, culture supernatants of *Porphyromonas gingivalis* efficiently degrades α- and β-defensins (Carlisle et al., 2009) and other AMPs, but resistance to those peptides seems to be independent of the proteolytic activity (Bachrach et al., 2008).

Species of *Bacillus* that produce metalloproteases rapidly degrade LL-37 and are more resistant to this CAMP than non producing species. Culture supernatants of *B. anthracis* also degrade LL-37 and are shown to increase LL-37 resistance in *B.* *subtilis*, a hypersensitive species, when grown together (Thwaite et al., 2006). Another example of protease-mediated AMP resistance occurs in *Burkholderia cenocepacia*, which produces two zinc-dependent metalloproteases (ZmpA e ZmpB) that inactivate different AMPs (Kooi and Sokol, 2009). Other proteases seem to mediate the resistance to lactoferrin B in *E. coli* and *S. aureus* (Ulvatne, 2002), like DegP in *E. coli*, a protease that also acts as a heat-shock protein.

Some members of the omptins family of outer membrane proteases present in Gram-negative bacteria are reported to mediate and contribute to resistance to AMPs. The PgtE protease of *S. enterica* Serovar *typhimurium* and CroP protease of *Citrobacter rodention* promote resistance to α-helical peptides (Guina et al., 2000; Le Sage et al., 2009). OmpT has an important role in the resistance of *E. coli* to protamine and urinary cationic peptides (Stumpe et al., 1998; Hui et al., 2010). In enterohemorrhagic *E. coli* (EHEC), OmpT contributes to the degradation of LL-37 more efficiently than in enteropathogenic *E. coli* (EPEC). The difference between EHEC OmpT and EPEC OmpT in providing defense against AMPs is due to the differences in the regulation of *ompT* genes in a promotor dependent manner, resulting in high levels of *ompT* transcripts and OmpT protein in EHEC (Thomassin et al., 2012). A posterior study with uropathogenic *E. coli* (UPEC) demonstrated that UPEC OmpT can cleave and protect the membrane from LL-37, but only when is expressed in high levels (Brannon et al., 2013), similar to those reported by Thomassin et al. (2012) for EPEC. Therefore, UPEC OmpT is not essential to LL-37 resistance. These data suggest that the role of OmpT in resistance to AMPs is specific to each pathogen.

In order to avoid killing by AMPs, pathogens may form complexes that retain their own proteases near the bacterial surface (Nyberg et al., 2004). This study shows that the host proteinase inhibitor α_2_-macroglobulin (α_2-_M) binds with high affinity to GRAB, a protein attached to *S. pyogenes* cell wall. The α_2-_M, on the other hand, traps the SpeB protease secreted by the bacteria, but SpeB retains the ability to degrade LL-37 and, more interestingly, its proteolytic activity against that AMP molecule is enhanced.

### EFFLUX PUMPS

Efflux pumps are energy-dependent transporters that extrude toxic compounds, including antibiotics, being one of the major mechanisms by which microbial pathogens resist to different classes of antibiotics (Piddock, 2006; Poole, 2007). Kupferwasser et al. (1999) demonstrated that a naturally occurring plasmid containing *qacA* gene, which encodes a proton motive force-dependent multidrug efflux pump, confers resistance to tPMP-1 in *S. aureus*, while constructions with deletions that compromise the *qacA* gene abolish tPMP-1 resistance. The *qacA-*mediated resistance seems to be specific to tPMP-1, because the expression of *qacA* did not confer resistance to other AMPs. It is possible that the efflux function of QacA protein itself is not responsible for the resistance to tPMP-1, but the alterations on the cytoplasmic membrane of strains expressing *qacA*. On the other hand, in *Neisseria gonorrhoeae* and *N. meningitidis*, the energy-dependent Mrt efflux pump mediates the resistance to antibacterial peptides PG-1, PC-8, LL-37, and TP-1 (Shafer et al., 1998; Tzeng et al., 2005). Notably, this pump recognizes structurally unrelated AMPs, such as PG-1, which folds into a β-sheet conformation, and LL-37, that assumes n α-helical structure. The importance of Mrt efflux pump system to avoid AMPs-killing during *in vivo* infections by *N. gonorrhoeae* was demonstrated by Jerse et al. (2003) where MtrCDE mutants were more susceptible to the host immune system. Warner et al. also showed that mutations upstream of *mrtC,* on the promoter of the *mtrR* gene encoding a repressor for MtrCDE, or in the binding site of MrtR, confer more resistance to LL-37 and CRAMP-8, a murine analogous to LL-37, by increasing the levels of MrtE and *mrtC*RNA stability (Warner et al., 2008). These mutants were also more fitted *in vivo* than wild-type strains, highlighting the importance of this pump in evasion of the immune system.

Resistance to polymyxin B in *Y. enterocolitica* and *K. pneumonia* involves a system composed of two proteins, RosA/RosB and AcrAB, respectively (Bengoechea and Skurnik, 2000; Padilla et al., 2010). The dissipation of the proton-motive force by the addition of Carbonyl cyanide *m*-chlorophenyl hydrazone CCCP reduced the resistance to polymyxin B, highlighting the importance of the energy-driven efflux pump of AMPs in resistance to these molecules. In *Y. enterocolitica,* RosA functions as a CAMP proton motive force-dependent efflux pump, while RosB has a potassium antiporter activity, involved in the regulation of intracellular pH, which is linked to CAMPs resistance by acidification of the cytoplasm. Both, RosA and RosB, are required for resistance to CAMPs and the presence of these defense molecules increases the expression of the *ros locus*, as observed in *S. pneumoniae* LL-37 resistance, mediated by MefE/Mel efflux pump (Zahner et al., 2010). Similarly, the protein TrkA of the potassium transport complex Trk is required for CAMP resistance in *Vibrio vulnificus* (Chen et al., 2004). It is hypothesized that protamine forms channels through which K^+^leaks from the cell; then, the high-rate K^+^uptake systems pump potassium inside the cell, preventing bacterial death until the protamin is degraded by extracellular proteases. Likewise, *sapG* mutants, a *S. typhimurium* gene that encodes a protein involved in potassium transport higlhy homologous to TrkA, exhibited hypersensitivity to protamin (Parra-Lopez et al., 1994). On the other hand, the overexpression of AcrAB, MexAB and NorA pumps in *E. coli*, *P. aeruginosa*, and *S. aureus*, respectively, did not increase resistance to CAMPs such HNP-1, HNP-3, HD-5, HBD-2, HBD-3, and LL-37, when compared to the wild-type strains and inactivated mutants. These results indicate that specific efflux pumps extrude specific types of AMPs (Rieg et al., 2009).

### TRAPPING

Some secreted molecules can bind to and neutralize the microbicidal activity of AMPs. For example, SIC proteins produced by *S. pyogenes* M1 bind to several AMPs, blocking their antimicrobial activity in the early stages of infection (Frick et al., 2003; Fernie-King et al., 2004; Pence et al., 2010). SIC binds more efficiently to LL-37 than to the defensing human neutrophil peptide HNP-1 and SIC deficient mutants are more sensitive to LL-37 than wild-type strains. Other SIC variants from other serotypes also bind and interfere with the antimicrobial activity from AMPs, but SIC from M1 strain is more potent (Frick et al., 2003). In addition to the plasminogen binding activity, Staphylokinase (SAK) from *S. aureus* can also binds to HNP-1 and HNP-2. SAK induces HNPs release from neutrophils, and the formation of a complex of SAK and the α-defensin results in the loss of antimicrobicidal activity of those defense molecules. This complex abrogates bactericidal activity of HNPs in other species, when grown together.

Other trapping molecules can be found attached to the bacterial surface, like M1 protein from *S. pyogenes.* M1 mutant strains are killed more efficiently by cathelicidins, LL-37 or mCRAMP than the wild-type strain. It seems that M1 acts as a shield, binding, and trapping the AMPs before they can reach the cell membrane and kill the pathogen (Lauth et al., 2009). Pili also may be a trap to AMPs. Maisey et al. (2008) reported that PilB from Group B *S.* sequesters LL-37 and mCRAMP, increasing the resistance to those peptides. GRABs also mediate an important binding mechanism in resistance to LL-37 by *S. pyogenes* (Nyberg et al., 2004).

It is also described that the shedding of host molecules by proteases from pathogens are involved in sequestration and resistance to several AMPs. For example, extracellular proteases secreted by *P. aeruginosa*, and *E. faecalis* degrade decorin proteoglycan, producing dermatan sulphate that protects those bacteria from killing mediated by HPN-1 through directly binding to the α-defensin (Schmidtchen et al., 2001).

## CONCLUSION

The increasing emergence of antibiotic resistance among human pathogens has led to the search for alternatives to overcome this alarming problem. Despite their ancient origin, only in the past decades the study of AMPs have gained increased attention as a promising therapeutic alternative against pathogenic microorganisms due their broad spectrum of activity against several species of bacteria, fungi, protozoa, and enveloped virus. Although the main target of AMPs was previously thought to be bacterial membrane several new and frequently, combined strategies of killing have been described in the past decades. Resistance to these molecules seems to be less common than to conventional antibiotics but was selected in several pathogens during the co-evolution of host and pathogens. A better comprehension of AMPs mode of action and counterpart resistance mechanisms is fundamental for the design of optimized AMPS that could be efficiently used as therapeutic drugs.

## Conflict of Interest Statement

The authors declare that the research was conducted in the absence of any commercial or financial relationships that could be construed as a potential conflict of interest. The editor and authors declare that while the editor shares the same institution and department as the authors, there has been no conflict of interest during the review and handling of this manuscript.
